# The role of resveratrol in bone marrow‐derived mesenchymal stem cells from patients with osteoporosis

**DOI:** 10.1002/jcb.28922

**Published:** 2019-05-20

**Authors:** Jing Li, Zhaoxu Xin, Mingjun Cai

**Affiliations:** ^1^ Drug Clinical Trial Institution Office The Third Affiliated Hospital of Qiqihar Medical University Qiqihar China; ^2^ Department of Orthopedics The Third Affiliated Hospital of Qiqihar Medical University Qiqihar China; ^3^ State Key Laboratory of Electroanalytical Chemistry, Changchun Institute of Applied Chemistry Chinese Academy of Sciences Changchun Jilin China

**Keywords:** Resveratrol, Bone marrow mesenchymal stem cells, Proliferation, Differentiation, Apoptosis

## Abstract

The aim of the present study was to investigate the effects of resveratrol on BMSCs from patients with osteoporosis. The cell viability and proliferation of BMSCs after treatment with different concentrations of resveratrol was respectively observed by MTT assay and EdU staining. The apoptosis was assessed using by TUNEL staining and the pluripotency was analyzed by quantitative reverse transcription‐PCR (qRT‐PCR). The osteogenic differentiation and adipogenic differentiation were determined by alkaline phosphatase (ALP) staining, alizarin red S (ARS) staining, oil red O (ORO) staining and qRT‐PCR analysis. MTT assay showed that Res at 40, 80, 100 μM markedly improved the cell proliferation of BMSCs from patients with osteoporosis. EdU staining indicated that Res treatment significantly accelerated the proliferation of BMSCs. In addition, the results of TUNEL staining revealed that Res at 40, 80, 100 μM inhibited the osteoporosis‐related apoptosis of BMSCs. qRT‐PCR analysis explored that Res treatment played a positive role in the pluripotency in BMSCs. ALP, ARS staining and qRT‐PCR demonstrated that Res promoted the differentiation of BMSCs into osteoblasts, especially at 80 μM. ORO staining and qRT‐PCR analysis proved that treatment of Res inhibited the adipogenesis of BMSCs isolated from patients with osteoporosis. Our findings suggested that Res can play a vital role in the cell viability, proliferation, apoptosis, pluripotency, osteogenesis and adipogenesis of BMSCs. And Res might be an efficient therapeutic approach for treating patients with osteoporosis.

## Graphical abstract

Our findings suggested that resveratrol (Res) can play a vital role in the cell viability, proliferation, apoptosis, pluripotency, osteogenesis and adipogenesis of bone marrow‐derived mesenchymal stem cells. And Res might be an efficient therapeutic approach for treating patients with osteoporosis.

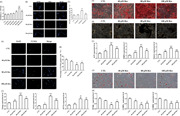

## 1 INTRODUCTION

Osteoporosis (OP), which is known as one of chronic inflammatory bone disease, is the clinical consequences of disruptions in skeletal homeostasis.^1^ OP is characterized by bone microarchitectural deterioration, bone loss, and remarkable decrease in loss of bone mineral density, accompanied by increase in the risk of bone fracture.^2^ This disease is commonly observed on a global scale and become a major public health challenge worldwide.^3^ OP results from an imbalance between osteoclast‐mediated bone resorption and osteoblast‐related bone formation.^4^ Recently, new therapeutic methods are used for treatment of OP, aiming at decreasing bone resorption by osteoclasts and/or promoting bone formation by osteoblasts.^5^ In clinical practice, several types of drugs are used for treating OP, such as bisphosphonates, vitamin D analogues, estrogen and calcitonin, which are regarded as bone absorption‐inhibitor drugs that can reverse the bone mass of patients with OP by suppressing osteoclast activity.^6^ However, these drugs just exhibit modest roles in increasing bone mass and are not able to increase the bone‐forming ability. According to prior studies, osteoblasts play a vital role in the pathogenesis of OP by taking a direct role in bone remodeling and bone regeneration.^7^ Therapies which can improve bone‐forming ability are more important in curing OP and finding novel therapeutic agents is urgently needed and of great significance.

Bone marrow‐derived mesenchymal stem cells (BMSCs, also known as bone marrow skeletal stem cells) are a type of adult stem cells, which reside in the perivascular compartment of bone marrow, but appear to be present in many tissues.^8^ BMSCs have the self‐renewal capacity and multidirectional differentiation potential, and they can differentiate into multiple cell lineages, including osteoblasts, chondrocytes, and adipocytes under specific conditions.^9^ They also possess the immune‐modulatory functions and can secret some paracrine factors which participate in endogenous tissue regenerative capacity. Current studies suggest that these characteristics make BMSC an ideal cell source for tissue engineering of cell‐based therapies for many degenerative diseases including OP.^10^ Some reports have showed that BMSCs are the main source of osteoblasts and osteocytes which play an important role in bone microenvironment.^11^ Therefore, inducing BMSCs to differentiate into osteoblasts is of great importance to treating bone disease and bone regeneration.

Resveratrol (*trans*‐3,4,5′‐trihydroxystilbene, Res) is a kind of nonflavonoid polyphenolic compound and naturally found in various plants, such as grapes, nuts and berries.^12^ It is usually generated in response to stress, injury and fungal infection and is able to serve as estrogens by binding to estrogen receptors, with health benefits for various diseases.^13^, ^14^, ^15^ Besides, resveratrol has multiple bioactivities of antioxidants, antiaging, insulin sensitization and plays a critical role in many diseases.^16^, ^17^, ^18^ Several reports have showed that resveratrol was capable of decreasing the levels of blood glucose in diabetic rats, normal rats and mice and humans.^19^, ^20^ Resveratrol can interact with multiple molecular targets which are related to inflammation and immunity. A lot of evidence reveal resveratrol exhibits the antitumor functions during certain processes during cancer initiation, angiogenesis, apoptosis, and metastasis.^21^ However, whether resveratrol plays a role in the biological functions of BMSCs and OP is yet unknown and need to be clearly elucidated.

The purpose of our study is to investigate the roles of resveratrol in patients with OP and detect the effects of Res on the biological behaviors of BMSCs at different concentrations. The results from this study uncovered that resveratrol can increase the cell viability and promote the proliferation of BMSCs, which might be decreased by OP. Functional analysis revealed that Res can decrease the apoptosis of BMSCs and upregulate the expression levels of pluripotency related genes. In addition, further analysis indicated that Res promoted the osteogenesis and inhibited the adipogenesis of BMSCs. Collectively, our findings uncovered that Res can be an efficient therapeutic approach for treating patients with OP.

## 2 MATERIALS AND METHODS

### 2.1 Clinical sample data and cell culture

Primary BMSCs were isolated from three osteoporotic patients, which were purchased from ScienCell (San Diego, CA). All the patients met the diagnostic standard of OP. All the patients did not receive any drug treatment before at least half year until the study. The patients with other joint diseases, such as gout, rheumatoid arthritis, and other bone diseases were excluded from the present study. Besides, the patients suffered from any systemic inflammation, autoimmune disease, or chronic malignant diseases were not included in our study.

BMSCs were isolated and cultured according to the previous reports. The cells were cultured in Dulbecco modified Eagle's medium (DMEM; Corning Incorporated Corporation, Corning City, NY) supplemented with 10% heat‐inactivated fetal bovine serum and 1% penicillin, in a 5% CO_2_ humidified incubator (Thermo Fisher Scientific lnc., Waltham, MA) at 37°C. Culture medium was changed every 2 days to maintain the normal growth of BMSCs. When the cells reached approximately 70% to 80% confluence, they were passaged into next passage using trypsin (Beyotime Biotechnology, Shanghai City, China). Usually, BMSCs at passages 3 to 5 were used for additional analysis. Each assay in our present study was performed in triplicate, and all the experiments were repeated three times using BMSCs from different participants, respectively.

### 2.2 Resveratrol treatment

Res, which was used in this study, was purchased from (Sigma‐Aldrich, Shanghai City, China). Res was dissolved in dimethyl sulfoxide (DMSO) (Tianjin Fuyu Fine Chemical Co Ltd, China) at 1 M, and was then diluted with media to 1 mM (containing 0.1% DMSO). The Res solution was protected from light. BMSCs were seeded at a density of 2 × 10^4^ cells/cm^2^ and treated with resveratrol at a concentration of 0, 5, 10, 20, 40, 80, and 100 μM. After 48 hours, the cells were used for further analysis to detect the role of Res in BMSCs.

### 2.3 Methylthiazolyl tetrazolium assay

The capacity for cell viability was measured using methylthiazolyl tetrazolium (MTT) assay. For the MTT analysis, BMSCs were trypsinized according to the procedures and plated in a 96‐well plate at a density of 5 × 10^3^ cells per well by using culture medium. The cells were treated with different concentrations of Res and the cell viability of cells was detected by performing MTT assay as described previously. Briefly, when the cells reached about 80%, 20 μL MTT solutions (5 μg/mL; Biasharp, Suzhou City, Jiangsu Province, China) were added to each well. Then the cells were incubated in MTT dye for 4 hours in a 37°C humidified incubator. After 4 hours incubation, the solution was discarded and then we added 100 to 150 μL volume of DMSO (Thermo Fisher Scientific) to each well for 15 minutes. Then, the plate was gently shaken for 15 minutes to mix the solutions. The results were analyzed using a microplate reader at 570 nm.

### 2.4 5‐Ethynyl‐2′‐deoxyuridine staining

The proliferation of BMSCs was determined using 5‐ethynyl‐2′‐deoxyuridine (EdU) staining which was performed as previously described.^22^ In brief, the cells were seeded in 24‐well plates using culture medium. After treatment with Res, the EdU staining was conducted with EdU Apollo kit (RiboBio, Guangzhou City, Guangdong Province, China) and 4′,6‐diamidino‐2‐phenylindole (DAPI) solution (DAPI:phosphate‐buffered saline [PBS] = 1:50; Solarbio, Peking City, China). In the end, 10 pictures were taken randomly by a microscope (Olympus, Tokyo, Japan). The proliferation capacity was analyzed by measuring the percentage of EdU‐positve cells in DAPI‐stained cells.

### 2.5 RNA isolation and quantitative reverse transcription‐PCR

Total RNA was extracted from BMSCs by using TRIzol reagent (Thermo Fisher Scientific). The RNA was reverse‐transcribed using the ABI reverse transcription kit (Applied Biosystems New York City, CA) according to standard techniques. quantitative reverse transcription polymerase chain reaction (qRT‐PCR) analysis was conducted using SYBR green and specific primers. PCR primers used in the study were designed and synthesized by GenePharma, China. A cycling program was as follows: 94°C for 2 minute for 1 cycle, 92°C for 20 seconds, 68°C for 30 seconds, and 72°C for 12 cycles with a decrease in 1°C per cycle and then at 92°C for 20 seconds, 57°C for 30 seconds, and 72°C for 20 seconds for 20 to 25 cycles. The expression of target genes was normalized to glyceraldehyde 3‐phosphate dehydrogenase (GAPDH) using the $2^{-\Delta \Delta C_{t}}$ method. The sequences of the genes were listed as following: Nanog‐F (5′‐TCTCTCAGGCCCAGCTGTGT‐3′), Nanog‐R (5′‐GCTTGCACTTCATCCTTTGGTT‐3′), Sox2‐F (5′‐ACCAGCTCGCAGACCTACAT‐3′), Sox2‐R (5′‐CCTCGGACTTGACCACAGAG‐3′), Oct4‐F (5′‐CCCGGAAGAGAAAGCGAACT‐3′), Oct4‐R (5′‐AGAACCATACTCGAACCACATCCT‐3′), ALP‐F (5′‐ACAACCTGACTGACCCTTCG‐3′), ALP‐R (5′‐TCATGATGTCCGTGGTCAAT‐3′), BMP4‐F (5′‐TCGTTACCTCAAGGGAGTGG‐3′), BMP4‐R (5′‐ATGCTTGGGACTACGTTTGG‐3′), Osterix‐F (5′‐AGAGGTTCACTCGCTCTGACGA‐3′), Osterix‐R (5′‐TTGCTCAAGTGGTCGCTTCTG‐3′), PPARγ‐F (5′‐TCACAAGAGGTGACCCAATG‐3′), PPARγ‐R (5′‐ CCATCCTTCACAAGCATGAA‐3′), C/EBPα‐F (5′‐GTGTGCACGTCTATGCTAAACCA‐3′), C/EBPα‐R (5′‐ GCCGTTAGTGAAGAGTCTCAGTTTG‐3′), C/EBPβ‐F (5′‐CATCACTGCCACCCAGAAGAC‐3′), C/EBPβ‐R (5′‐CCAGTGAGCTTCCCGTTCAG‐3′), GAPDH‐F (5′‐CATCACTGCCACCCAGAAGAC‐3′), GAPDH‐R (5′‐CCAGTGAGCTTCCCGTTC AG‐3′).

### 2.6 Terminal deoxynucleotidyl transferase dUTP nick‐end labeling staining

To measure the apoptosis levels of BMSCs, about 1 × 10^5^ cells were plated in a small dish. The cells treated with Res were used for terminal deoxynucleotidyl transferase dUTP nick‐end labeling (TUNEL) staining. The culture medium in the dish was discarded, and the cells were fixed in 4% Paraformaldehyde (PFA) (Beyotime) after washed with PBS (Beyotime). After the closure and penetration procedures, TUNEL staining solution (Roche, Basel City, Switzerland) Vial1 and Vial2 were mixed in a ratio of 1:9 according to the instructions of the kit. Then, cells were incubated by the mixture for 1 hour in a dark room. After washed with PBS, cells were incubated with DAPI solution (Solarbio) for 30 minutes. Finally, the cells were observed under a fluorescence microscope (Nikon Corporation, Tokyo City, Japan).

### 2.7 Osteogenic differentiation and alizarin Red S staining

To induce BMSCs into osteoblasts, the cells were cultured in a 24‐well plate by using osteogenic induction medium for 10 days. The osteogenic induction medium was composed of DMEM/F12, 0.01 mM 1,2,5‐dihydroxyvitamin D3, 50 mM ascorbate‐2‐phosphate, and 10 mM β‐glycerophosphate. BMSCs were cultured in 24‐well plates and induced into osteoblasts to examine the deposited mineral. The cells were induced for 10 days and then washed three times with PBS and fixed using 1 mL volume of 4% PFA at room temperature for 30 minutes. The plates were then rinsed twice using double‐distilled water (ddH_2_O) and then incubated with alizarin red S (ARS) staining solution (Cyagen, Guangzhou City, Guangdong Province, China) at room temperature for about 30 minutes. Finally, cells were gently washed by ddH_2_O, and images were observed via a microscope.

### 2.8 Alkaline phosphatase staining

BMSCs were cultured in osteogenic induction medium for 10 days and the cells were used to perform alkaline phosphatase (ALP) staining. First, the medium was removed and the cells were fixed using 4% PFA. Then, the cells were stained with ALP staining solution (Nanjing Jiancheng, Nanjing City, Jiangsu Province, China) for 30 minutes, followed by several washes with PBS. The pictures were captures under a microscope (Nikon Corporation).

### 2.9 Adipogenic differentiation and oil Red O staining

To induce BMSCs into adipocytes, the cells were treated with DMEM medium consisting of 0.5 mM 3‐iso‐butyl‐1‐methylxanthine, 1 mM dexamethasone, and 5 μM insulin for 20 days. The oil Red O (ORO) stock solution (Cyagen) was prepared before the experiments and mixed with PBS at a ratio of 3/2 to make the working solution. BMSCs which cultured in adipogenic induced medium were washed by PBS, fixed in 4% PFA for 30 minutes at room temperature. Then, the cells were rinsed with water and treated with ORO working solution for 15 minutes. In the end, the cells were observed and imaged using bright‐field microscopy.

### 2.10 Statistical analysis

All data are presented as the mean ± standard deviation (SD). Data between the two groups were analyzed using *t* tests. Statistical difference was analyzed by one‐way analysis of variance using GraphPad Prism 5 software (GraphPad Software, La Jolla, CA). *P*  < 0.05 were considered statistically significant differences.

## 3 RESULTS

### 3.1 The effects of res on the proliferation of BMSCs from osteoporotic patients

**Figure 1:**
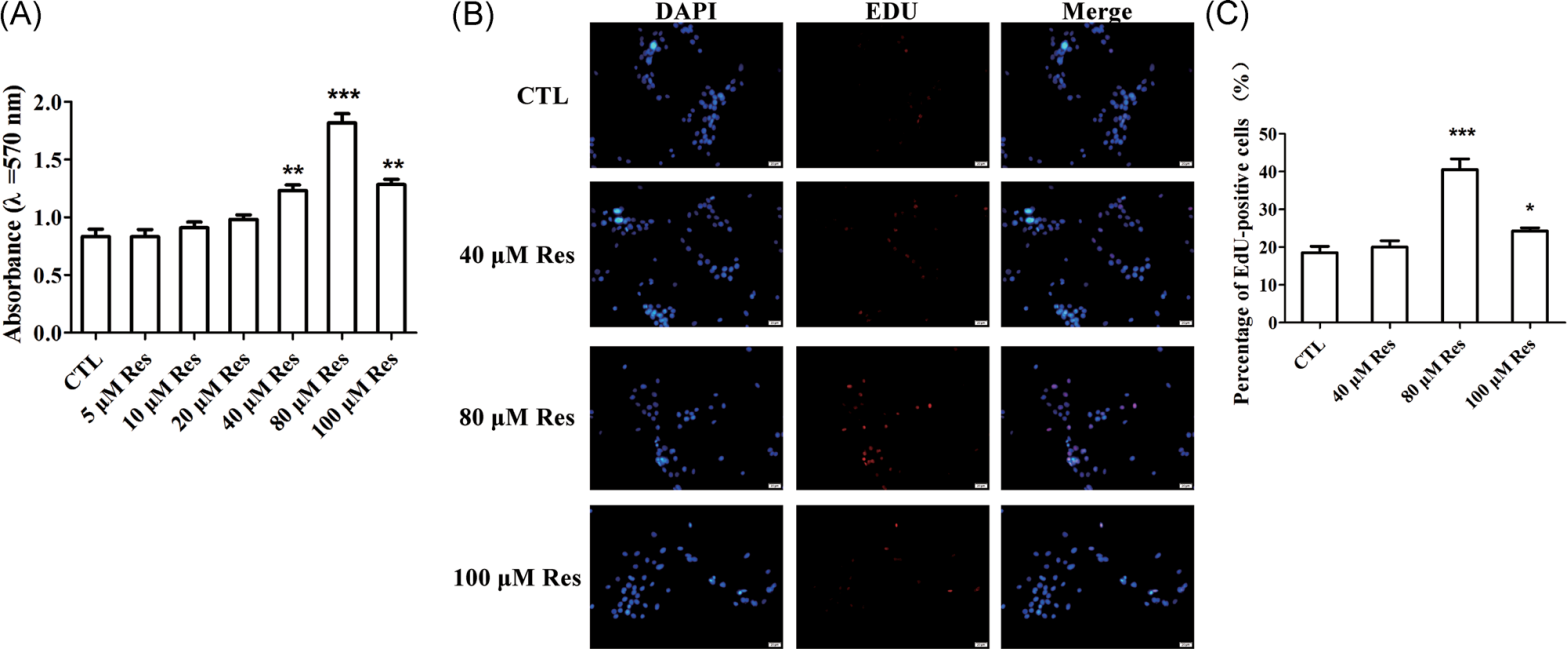
Res induced the increase in the cell viability and proliferation of BMSCs. A, The cell viability of BMSCs from patients with osteoporosis after Res treatment was detected by MTT assay. B, EdU staining determined the effect of Res on the proliferation of BMSCs derived from osteoporotic patients. C, Quantification analysis of EdU staining. n = 3 per group. BMSC, bone marrow‐derived mesenchymal stem cell; EdU, 5‐ethynyl‐2′‐deoxyuridine; MTT, methylthiazolyl tetrazolium; Res, resveratrol. Data are shown as mean ± SD. *P < 0.05, **P < 0.01, and ***P < 0.001

First, we investigated whether Res treatment affected the viability and proliferation of BMSCs isolated from osteoporotic patients. The cells were cultured in growth medium and treated with Res at various concentrations for 24 hours. After treatment with Res at a concentration of 0, 5, 10, 20, 40, 80, and 100 μM, the cell proliferation was gradually increased (Figure 1A). Among different concentrations, Res at 40, 80, and 100 μM significantly enhanced the cell proliferation of BMSCs from patients with OP and other concentrations of Res played no effects on the cell viability of BMSCs (Figure 1A). And the absorbance of BMSCs peaked after treatment of 80 μM Res. Therefore, we chose Res at 40, 80, and 100 μM for further analysis. In addition, we further detected the role of Res in the proliferation of BMSCs using EdU staining. The result of EdU staining showed that 80 μM Res obviously promoted the proliferation of BMSCs from osteoporotic patients compared with nontreated cells (Figure 1B,C). And Res at 40 and 100 μM also facilitated the proliferation of BMSCs (Figure 1B,C). The above results suggested that Res could stimulate the proliferation of BMSCs from patients with OP.

### 3.2 The roles of Res in the apoptosis and pluripotency of BMSCs from osteoporotic patients

**Figure 2:**
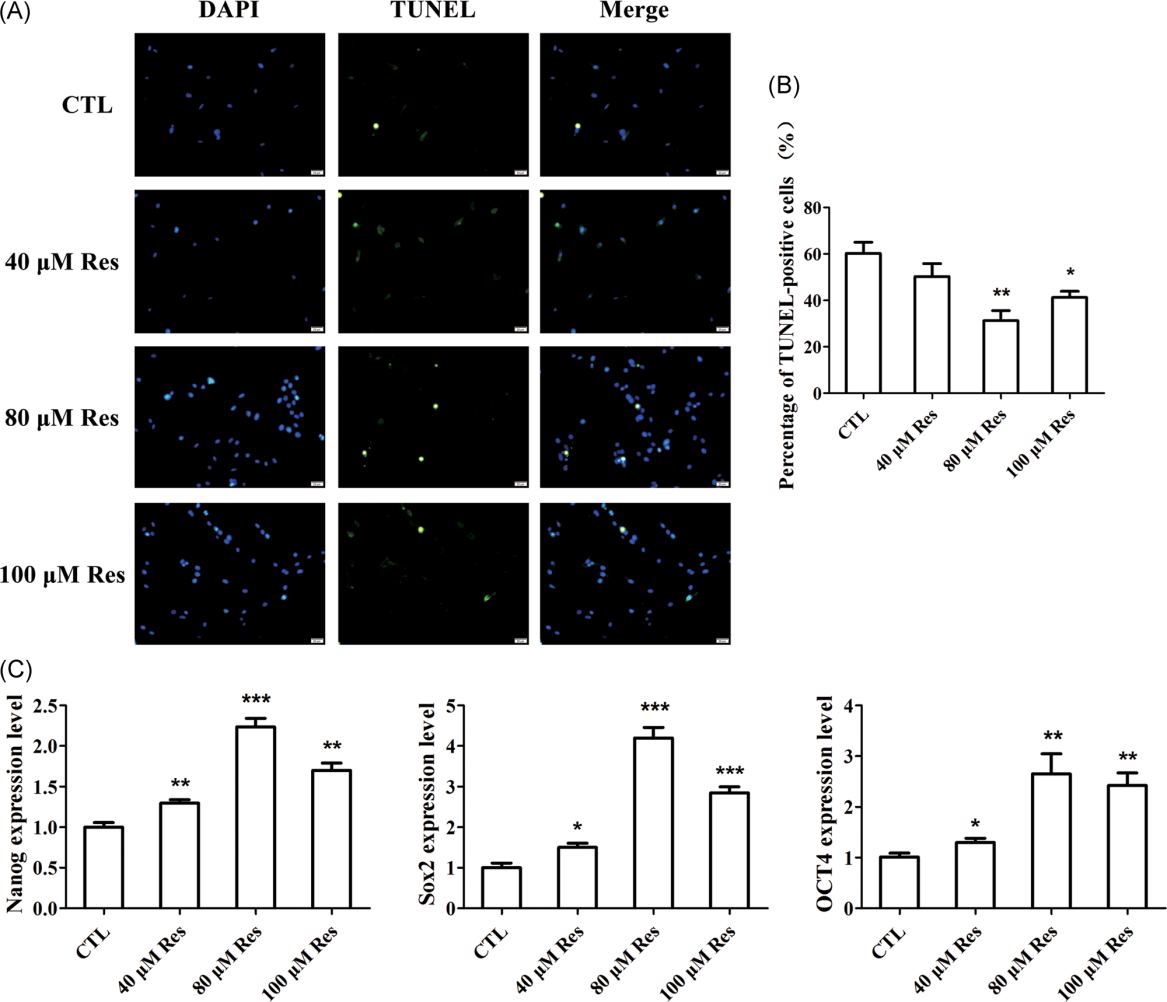
Res inhibited the apoptosis but promoted the pluripotency of BMSCs. A, Images of EdU staining of BMSCs after treatment with different concentrations of Res. B, Quantification analysis of TUNEL staining. C, qRT‐PCR analysis measured the role of Res in the pluripotency of BMSCs. n = 3 per group. Data was representative of three independent experiments. n = 3 per group. Data are shown as mean ± SD. BMSC, bone marrow‐derived mesenchymal stem cell; EdU, 5‐ethynyl‐2′‐deoxyuridine; qRT‐PCR, quantitative reverse transcription polymerase chain reaction; Res, resveratrol; TUNEL, terminal deoxynucleotidyl transferase dUTP nick‐end labeling. *P < 0.05, **P < 0.01, and ***P < 0.001

To confirm the effect of Res on the apoptosis of BMSCs from patients with OP, we treated the cells with Res at a concentration of 40, 80, and 100 μM. After 24 hours, the cells were prepared for TUNEL staining. TUNEL staining indicated that Res decreased the number of apoptotic cells compared with control group (Figure 2A,B). Among the concentration of 40, 80, and 100 μM, Res at 80 μM obviously inhibited the apoptosis of BMSCs (Figure 2A,B). We further examined the effect of Res on the expression of pluripotent genes, such as Nanog, Sox2, and OCT4 in BMSCs of osteoporotic patients. As shown in Figure 2C, Res‐treated BMSCs exhibited the increase in the messenger RNA (mRNA) expression of Nanog, Sox2, and OCT4 (Figure 2C). In particularly, the expression of these genes in BMSCs after treated Res at 80 μM was markedly increased (Figure 2C). It suggested that Res treatment increased the pluripotency of BMSCs. The results proved that Res inhibited the apoptosis but increase the pluripotency of BMSCs from patients with OP.

### 3.3 Res reversed OP‐induced decrease of osteogenic differentiation of BMSCs

**Figure 3:**
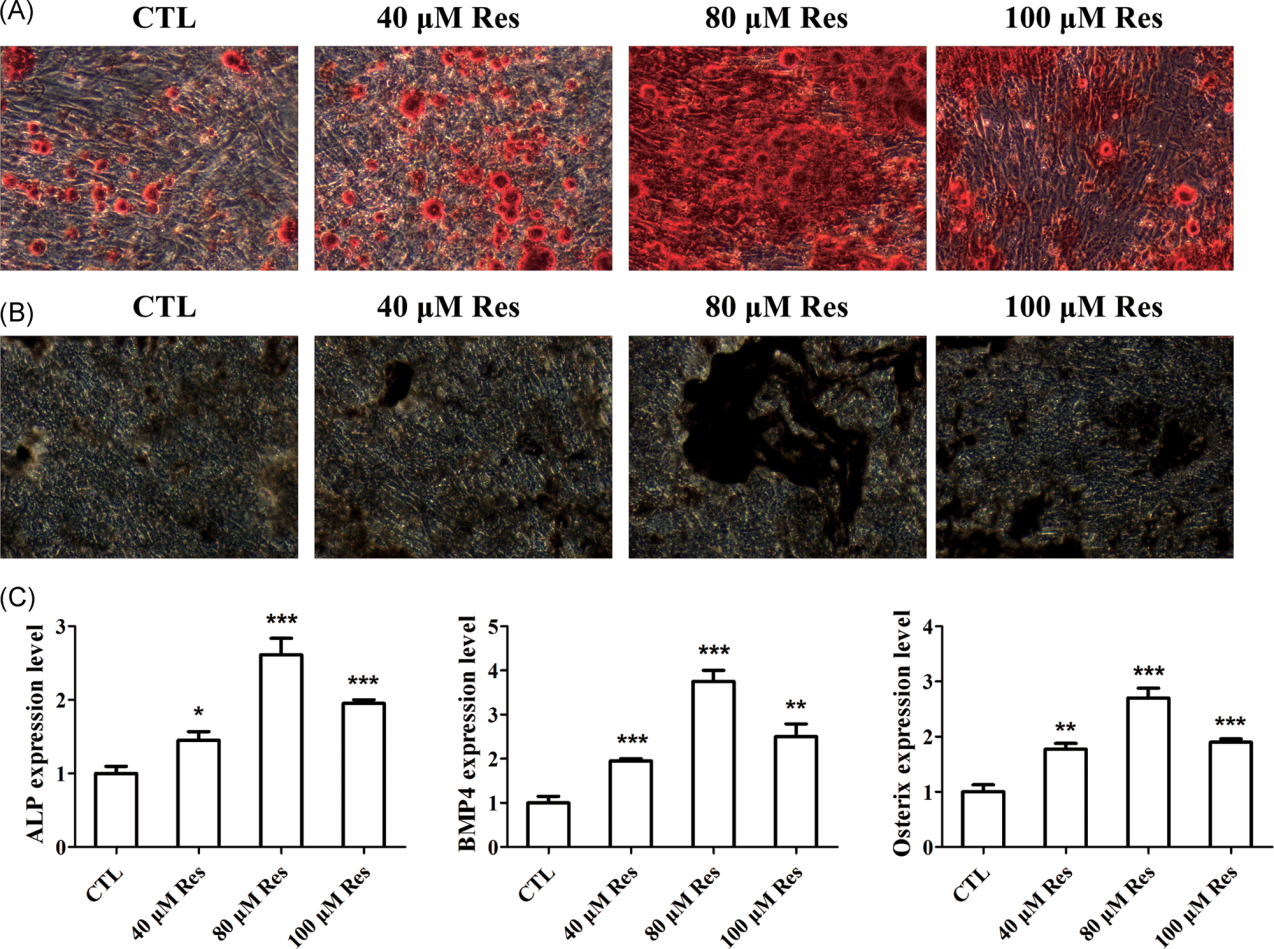
Res improved the potential of osteogenic differentiation in BMSCs. A,B, Representative images of ALP and ARS staining of matrix mineralization in BMSCs treated with different concentrations of Res after osteogenic differentiation of 10 days. C, qRT‐PCR analysis of the relative levels of osteoblast‐related genes in BMSCs treated with Res after osteogenic differentiation. n = 3 per group. Data are shown as mean ± SD. ALP, alkaline phosphatase, ARS, alizarin red S; BMP4, bone morphogenetic protein 4; BMSC, bone marrow‐derived mesenchymal stem cell; qRT‐PCR, quantitative reverse transcription polymerase chain reaction; Res, resveratrol. *P < 0.05, **P < 0.01, and ***P < 0.001

To further explore the role of Res in the biological functions, we detected the osteogenic differentiation capacity of BMSCs of humans. ARS staining showed that Res improved the mineralization capability of BMSCs from osteoporotic patients, especially at concentration of 80 μM (Figure 3A). The results of ALP staining revealed that Res could notably accelerated the formation of mineralized nodules, which suggested that Res treatment played a stimulative role in osteogenesis of BMSCs (Figure 3B). Similarly, qRT‐PCR analysis demonstrated that Res significantly upregulated the mRNA expression of osteoblast‐specific genes, including ALP, bone morphogenetic protein 4 (BMP4), and osterix, which were important marker genes during the osteogenic differentiation of BMSCs (Figure 3C). These results indicated that Res positively regulated the differentiation of BMSCs into osteoblasts.

### 3.4 Res suppressed OP‐induced increase of adipogenic differentiation of BMSCs

**Figure 4:**
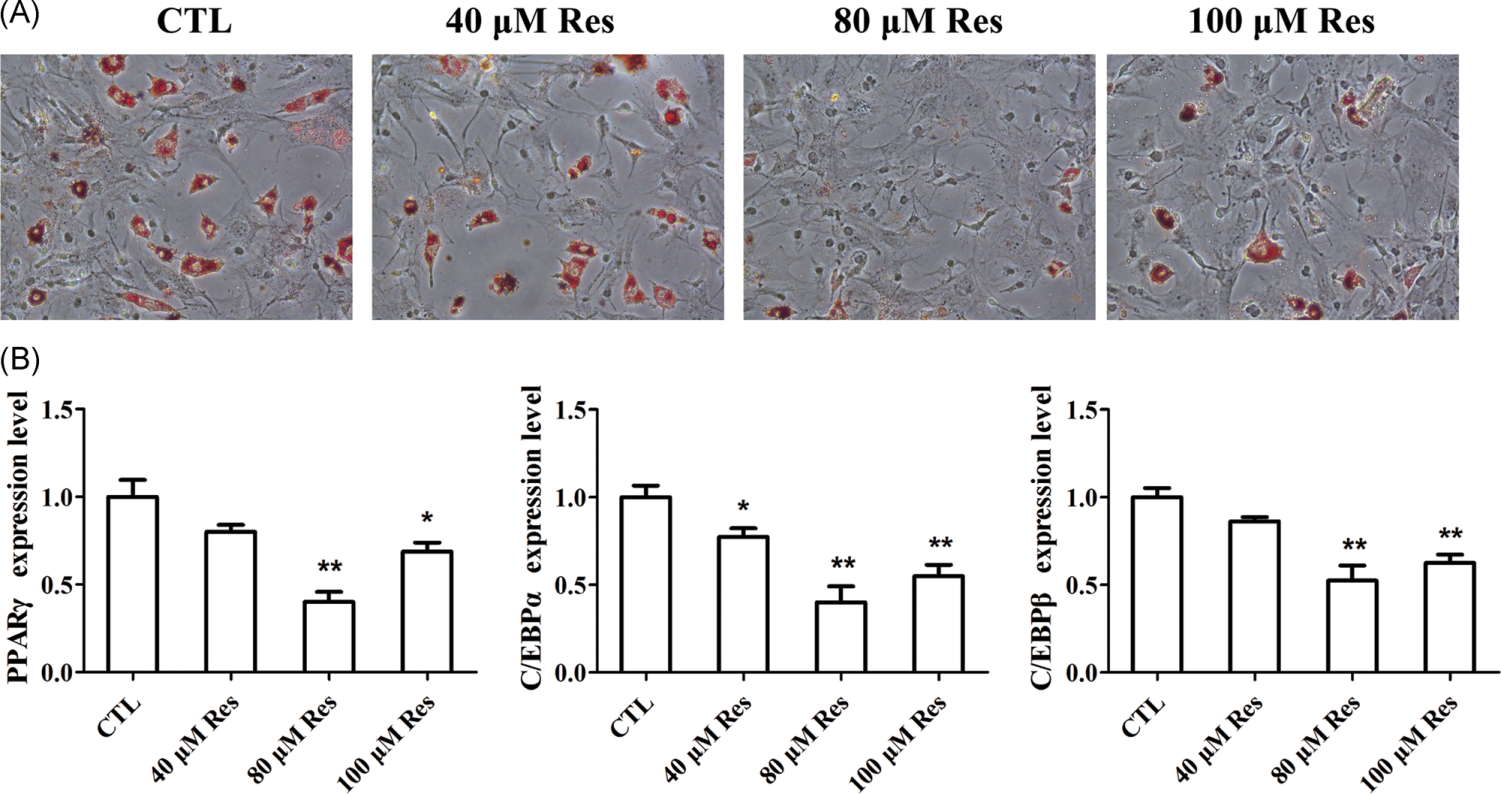
Res impaired the capacity of adipogenic differentiation in BMSCs. A, Oil Red O staining was performed to detect the number of lipids and the number of spots in BMSCs treated with Res after osteogenesis of 20 days. B, qRT‐PCR analysis of the relative mRNA expression levels of adipocyte marker genes in BMSCs treated with Res. n = 3 per group. Data are shown as mean ± SD. BMSC, bone marrow‐derived mesenchymal stem cell; C/EBPα, CCAAT/enhancer‐binding protein α, C/EBPβ, CCAAT/enhancer‐binding protein β; mRNA, messenger RNA; PPARγ, peroxisome proliferator‐activated receptor γ; qRT‐PCR, quantitative reverse transcription polymerase chain reaction; Res, resveratrol. *P < 0.05, **P < 0.01, and ***P < 0.001

To investigate whether Res regulated the adipogenesis of BMSCs from patients with OP, we applied the ORO staining and qRT‐PCR analysis. ORO staining revealed that Res at 40, 80, 100 μM dramatically decreased the number of oil drops and inhibited the formation of adipocytes, which indicating the Res impaired the adipogenesis of BMSCs (Figure 4A). Besides, the mRNA expression levels of adipocyte related genes, including peroxisome proliferator‐activated receptor γ (PPARγ), CCAAT/enhancer‐binding protein α (C/EBPα), CCAAT/enhancer‐binding protein β (C/EBPβ), were decreased by Res treatment (Figure 4B). The above results uncovered that Res led to the decrease in the adipogenic differentiation of BMSCs.

## 4 DISCUSSION

The present study firstly revealed that Res could increase the cell viability, proliferation and pluripotency as well as promoted the osteogenic differentiation of BMSCs, while Res treatment significantly inhibited the apoptosis and adipogenic differentiation of BMSCs from osteoporotic patients. These findings provided experimental foundation for Res as a novel treatment for BMSCs injury and bone diseases, including OP. Identification of the potential therapeutic methods responsible for OP might be conducive to therapeutic efforts.

Aging gives rise to many degenerative skeletal diseases, including OP and other bone diseases.^23^ OP, a systemic and multifactorial bone disease resulting in morbidity and mortality among the elderly, is increased year‐by‐year.^24^ Because of fragility, bone fractures become the most common clinical symptom of OP, thus making the elderly at risk for chronic pain and having difficulty of moving.^25^ In addition, fragility fractures bring about enormous social, economic, and individual burdens because of prolonged hospitalizations and medical treatments.^26^ The development of OP involves a balanced stem cell differentiation and a dysfunction of BMSCs.^27^ The function and balance of BMSCs are important to the bone development and bone microenvironment.

BMSCs exhibit degenerative changes, including imbalanced differentiation and reduced proliferation in the process of OP, which resulted in OP‐related bone loss and imbalance of bone microenvironment. BMSCs have been deemed as the main source of osteoblasts for bone development. The previous reports showed that degenerative changes of BMSCs from humans and rodents in the process of aging are related to bone aging.^28^ BMSCs are likely to lose their self‐renewal capacity and easy to differentiate into adipocytes rather than osteocytes in patients with OP, which results in bone loss and fat accumulation.^29^, ^30^

It has been demonstrated that Res has potential to decrease bone loss, which is caused by different etiologies and pathologies.^31^ For example, Res, acting as Notch activator, can be used as promising therapeutic molecules for promoting anabolic bone formation in patients with bone loss diseases.^32^ However, the role of Res in the human BMSCs has not been investigated. In our study, MTT assay showed that Res at 40, 80, and 100 μM markedly improved the cell proliferation of BMSCs from patients with OP. EdU staining indicated that Res significantly accelerated the proliferation of BMSCs, especially at concentration of 80 μM. In addition, the results of TUNEL staining revealed that Res inhibited the apoptosis of BMSCs. qRT‐PCR analysis explored that Res treatment played a positive role in the pluripotency in BMSCs. According to the previous study, Res elevates the osteogenic function of senescent BMSCs, suggesting that Res exhibits an antiosteoporotic role in senescence‐accelerated mice.^33^ In our study, ALP, ARS staining, and qRT‐PCR demonstrated that Res could positively regulate the osteogenic differentiation of BMSCs and promoted the differentiation of BMSCs into osteoblasts. ORO staining and qRT‐PCR analysis proved that treatment of Res inhibited the adipogenesis of BMSCs. Thus, we explored that Res protects BMSCs against apoptosis and adipogenic differentiation. And Res plays a vital role in the biological functions of BMSCs, including cell viability, proliferation, pluripotency, and osteogenic differentiation. However, we have not explored the mechanism of the roles of Res in BMSCs. In the further experiments, we will continue to investigate the underlying mechanism.

In conclusion, the results of our present study demonstrated that Res has the capacity of reversing OP‐induced decrease of cell viability, proliferation, pluripotency and osteogenesis in BMSCs isolated from patients with OP, and at the same time suppressing the OP‐induced apoptosis and adipogenesis of BMSCs. These findings help us toward a potential and novel therapeutic method for treating OP‐induced BMSCs dysfunction and related bone diseases.

## CONFLICT OF INTERESTS

All the authors declared no potential conflicts of interest with respect to the research, authorship, and publication of this article. Jing Li and Mingjun Cai designed the project and wrote the manuscript. Jing Li and Zhaoxu Xin finished the experiments.
